# 2-Amino-4-(3-fluoro­phen­yl)-6-(naphthalen-1-yl)pyridine-3-carbonitrile

**DOI:** 10.1107/S1600536810048257

**Published:** 2010-11-24

**Authors:** Jia-Ying Xu, Zhao-Chang Gao, Kai-Jin Sun, Wei-Hua Cheng

**Affiliations:** a College of Chemical and Biological Engineering, Yancheng Institute of Technology, Yinbing Road No. 9 Yancheng, Yancheng 224051, People’s Republic of China; b Department of Chemical Engineering, Yancheng College of Textile Technology, Yancheng 224051, People’s Republic of China

## Abstract

There are two independent mol­ecules in the asymmetric unit of the title compound, C_22_H_14_FN_3_, which differ slightly in the relative orientations of the naphthyl and phenyl groups with respect to the pyridyl ring framework. In one mol­ecule, the naphthyl ring system and the phenyl ring form dihedral of angles 56.50 (2) and 48.23 (3)°, respectively, with the pyridyl ring plane. In the other mol­ecule, the corresponding dihedral angles are 50.01 (2) and 51.1 (3)°, respectively. In the crystal, inter­molecular N—H⋯N hydrogen bonds connect the independent mol­ecules into dimers.

## Related literature

For general background to the use of the title compound an inter­mediate, see: Moreau *et al.* (1999 ▶). For the synthetic procedure, see: Mantri *et al.* (2008 ▶). For related structures, see: Mkhalid *et al.* (2006 ▶).
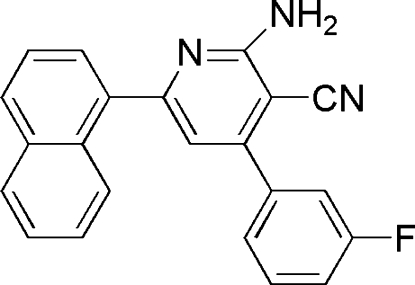


## Experimental

### 

#### Crystal data

C_22_H_14_FN_3_
*M* *_r_* = 339.36
Triclinic, 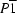

*a* = 11.750 (2) Å
*b* = 12.703 (3) Å
*c* = 13.457 (3) Å
α = 73.33 (3)°
β = 86.82 (3)°
γ = 63.98 (3)°
*V* = 1723.4 (8) Å^3^
*Z* = 4
Mo *K*α radiation
μ = 0.09 mm^−1^
*T* = 293 K
0.30 × 0.20 × 0.10 mm

#### Data collection

Enraf–Nonius CAD-4 diffractometer
Absorption correction: ψ scan (North *et al.*, 1968 ▶) *T*_min_ = 0.975, *T*_max_ = 0.991
6662 measured reflections
6327 independent reflections
3964 reflections with *I* > 2σ(*I*)
*R*_int_ = 0.021
3 standard reflections every 200 reflections  intensity decay: 1%

#### Refinement

*R*[*F* ^2^ > 2σ(*F* ^2^)] = 0.055
*wR*(*F* ^2^) = 0.161
*S* = 1.00
6327 reflections
470 parameters
H-atom parameters constrained
Δρ_max_ = 0.18 e Å^−3^
Δρ_min_ = −0.20 e Å^−3^

### 

Data collection: *CAD-4 Software* (Enraf–Nonius, 1985 ▶); cell refinement: *CAD-4 Software*; data reduction: *XCAD4* (Harms & Wocadlo, 1995 ▶); program(s) used to solve structure: *SHELXS97* (Sheldrick, 2008 ▶); program(s) used to refine structure: *SHELXL97* (Sheldrick, 2008 ▶); molecular graphics: *SHELXTL* (Sheldrick, 2008 ▶); software used to prepare material for publication: *SHELXTL*.

## Supplementary Material

Crystal structure: contains datablocks I, global. DOI: 10.1107/S1600536810048257/pv2356sup1.cif

Structure factors: contains datablocks I. DOI: 10.1107/S1600536810048257/pv2356Isup2.hkl

Additional supplementary materials:  crystallographic information; 3D view; checkCIF report

## Figures and Tables

**Table 1:** Hydrogen-bond geometry (Å, °)

*D*—H⋯*A*	*D*—H	H⋯*A*	*D*⋯*A*	*D*—H⋯*A*
N2—H2*A*⋯N4^i^	0.86	2.30	3.108 (3)	157
N5—H5*B*⋯N1^ii^	0.86	2.25	3.031 (3)	152

Symmetry codes: (i) 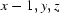; (ii) 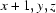.

**Fig. 1.**
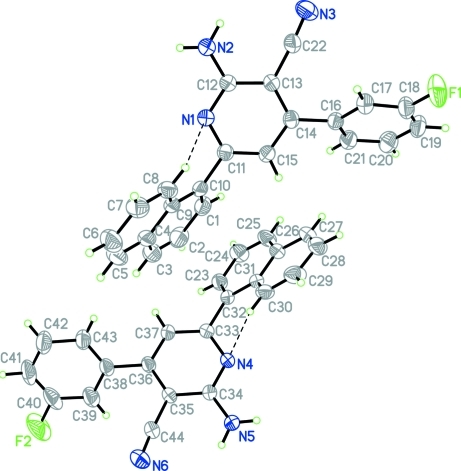
A perspective drawing of the two molecules of the title compound. Displacement ellipsoids are drawn at the 50% probability level.

**Fig. 2.**
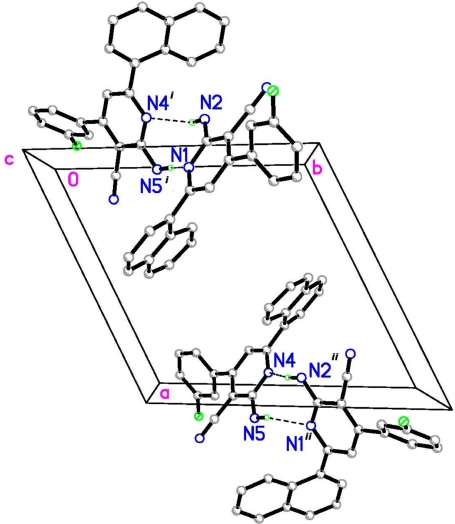
Unit cell packing of the title compound showing dimers formed via hydrogen bonding. H atoms not involved in hydrogen bonds have been omitted for clarity. Symmetry codes: (i) x - 1, y, z; (ii) x + 1, y, z.

## supplementary crystallographic information

### Comment

The title compound is an intermediate which can be used in many fields such as medicine (Moreau *et al.*, (1999). Herein we report its crystal structure. There are two independen tmolecules in an asymmetric unit of the title compound (Fig. 1), which differ slightly in the relative orientations of the naphthyl and phenyl groups with respect to the pyridyl ring framework. In one of the molecules, the naphthyl (C1—C10) and phenyl ring planes (C16 to C21) form torsion angles 56.50 (2) and 48.23 (3)°, respectively, with the middle pyridyl ring plane. In the other molecule, the corresponding torsion angles are 50.01 (2) and 51.1 (3)°, respectively. There are intermolecular N—H···N hydrogen bonds, which connect the independent molecules into dimers (Fig. 2 and Tab. 1). In addition, there are weak C—H···N intramolecular hydrogen bonds in each molecule.

### Experimental

The title compound was prepared by the literature method (Mantri, 2008). Crystals suitable for X-ray analysis were obtained by dissolving the title compound (0.5 g) in methanol (20 ml) and evaporating the solvent slowly at room temperature in about 7 d.

### Refinement

All H atoms were positioned geometrically and constrained to ride on their parent atoms, with C—H = 0.93 Å and N—H = 0.86 Å with *U*_iso_(H) = 1.2*U*_eq_(C/N).

### Crystal data

**Table:**

C_22_H_14_FN_3_	*Z* = 4
*M**_r_* = 339.36	*F*(000) = 704
Triclinic, *P*1	*D*_x_ = 1.308 Mg m^−^^3^
Hall symbol: -P 1	Mo *K*α radiation, λ = 0.71073 Å
*a* = 11.750 (2) Å	Cell parameters from 25 reflections
*b* = 12.703 (3) Å	θ = 10–13°
*c* = 13.457 (3) Å	µ = 0.09 mm^−^^1^
α = 73.33 (3)°	*T* = 293 K
β = 86.82 (3)°	Block, colourless
γ = 63.98 (3)°	0.30 × 0.20 × 0.10 mm
*V* = 1723.4 (8) Å^3^	

### Data collection

**Table:**

Enraf–Nonius CAD-4 diffractometer	3964 reflections with *I* > 2σ(*I*)
Radiation source: fine-focus sealed tube	*R*_int_ = 0.021
graphite	θ_max_ = 25.4°, θ_min_ = 1.6°
ω/2θ scans	*h* = 0→14
Absorption correction: ψ scan (North *et al.*, 1968)	*k* = −13→15
*T*_min_ = 0.975, *T*_max_ = 0.991	*l* = −16→16
6662 measured reflections	3 standard reflections every 200 reflections
6327 independent reflections	intensity decay: 1%

### Refinement

**Table:**

Refinement on *F*^2^	Secondary atom site location: difference Fourier map
Least-squares matrix: full	Hydrogen site location: inferred from neighbouring sites
*R*[*F*^2^ > 2σ(*F*^2^)] = 0.055	H-atom parameters constrained
*wR*(*F*^2^) = 0.161	*w* = 1/[σ^2^(*F*_o_^2^) + (0.087*P*)^2^] where *P* = (*F*_o_^2^ + 2*F*_c_^2^)/3
*S* = 1.00	(Δ/σ)_max_ < 0.001
6327 reflections	Δρ_max_ = 0.18 e Å^−^^3^
470 parameters	Δρ_min_ = −0.20 e Å^−^^3^
0 restraints	Extinction correction: *SHELXL97* (Sheldrick, 2008), Fc^*^=kFc[1+0.001xFc^2^λ^3^/sin(2θ)]^-1/4^
Primary atom site location: structure-invariant direct methods	Extinction coefficient: 0.019 (2)

### Special details

**Table:**

Geometry. All e.s.d.'s (except the e.s.d. in the dihedral angle between two l.s. planes) are estimated using the full covariance matrix. The cell e.s.d.'s are taken into account individually in the estimation of e.s.d.'s in distances, angles and torsion angles; correlations between e.s.d.'s in cell parameters are only used when they are defined by crystal symmetry. An approximate (isotropic) treatment of cell e.s.d.'s is used for estimating e.s.d.'s involving l.s. planes.
Refinement. Refinement of *F*^2^ against ALL reflections. The weighted *R*-factor *wR* and goodness of fit *S* are based on *F*^2^, conventional *R*-factors *R* are based on *F*, with *F* set to zero for negative *F*^2^. The threshold expression of *F*^2^ > σ(*F*^2^) is used only for calculating *R*-factors(gt) *etc*. and is not relevant to the choice of reflections for refinement. *R*-factors based on *F*^2^ are statistically about twice as large as those based on *F*, and *R*- factors based on ALL data will be even larger.

### Fractional atomic coordinates and isotropic or equivalent isotropic displacement parameters (Å^2^)

**Table:**

	*x*	*y*	*z*	*U*_iso_*/*U*_eq_	
F1	0.0789 (2)	0.84614 (19)	0.70934 (14)	0.0980 (7)	
N1	0.14789 (18)	0.52342 (18)	0.30964 (16)	0.0442 (5)	
N2	−0.0617 (2)	0.5757 (2)	0.33579 (19)	0.0648 (7)	
H2A	−0.0587	0.5252	0.3035	0.078*	
H2B	−0.1314	0.6168	0.3597	0.078*	
N3	−0.1749 (2)	0.7921 (2)	0.4670 (2)	0.0736 (8)	
C1	0.4154 (2)	0.5319 (3)	0.1941 (2)	0.0533 (7)	
H1B	0.3824	0.6163	0.1802	0.064*	
C2	0.5166 (3)	0.4717 (3)	0.1393 (2)	0.0657 (8)	
H2C	0.5491	0.5162	0.0892	0.079*	
C3	0.5665 (3)	0.3487 (3)	0.1598 (2)	0.0682 (9)	
H3A	0.6329	0.3096	0.1225	0.082*	
C4	0.5205 (2)	0.2784 (3)	0.2362 (2)	0.0526 (7)	
C5	0.5736 (3)	0.1498 (3)	0.2592 (3)	0.0718 (9)	
H5A	0.6404	0.1097	0.2229	0.086*	
C6	0.5285 (3)	0.0841 (3)	0.3335 (3)	0.0739 (9)	
H6A	0.5630	−0.0004	0.3466	0.089*	
C7	0.4306 (3)	0.1425 (3)	0.3905 (2)	0.0618 (8)	
H7A	0.4015	0.0964	0.4426	0.074*	
C8	0.3770 (2)	0.2665 (2)	0.37059 (19)	0.0486 (6)	
H8A	0.3121	0.3038	0.4097	0.058*	
C9	0.4182 (2)	0.3390 (2)	0.29187 (18)	0.0420 (6)	
C10	0.3645 (2)	0.4693 (2)	0.26713 (18)	0.0417 (6)	
C11	0.2517 (2)	0.5404 (2)	0.31648 (17)	0.0391 (6)	
C12	0.0431 (2)	0.5904 (2)	0.34890 (19)	0.0441 (6)	
C13	0.0410 (2)	0.6724 (2)	0.40287 (18)	0.0406 (6)	
C14	0.1493 (2)	0.6890 (2)	0.41083 (17)	0.0398 (6)	
C15	0.2547 (2)	0.6228 (2)	0.36471 (18)	0.0425 (6)	
H15A	0.3280	0.6335	0.3660	0.051*	
C16	0.1532 (2)	0.7746 (2)	0.46487 (18)	0.0419 (6)	
C17	0.1095 (2)	0.7728 (2)	0.56394 (19)	0.0496 (6)	
H17A	0.0743	0.7201	0.5964	0.060*	
C18	0.1196 (3)	0.8499 (3)	0.6121 (2)	0.0580 (7)	
C19	0.1671 (3)	0.9304 (3)	0.5684 (2)	0.0654 (8)	
H19A	0.1716	0.9821	0.6033	0.079*	
C20	0.2090 (3)	0.9333 (3)	0.4696 (3)	0.0675 (8)	
H20A	0.2413	0.9884	0.4373	0.081*	
C21	0.2032 (2)	0.8552 (2)	0.4187 (2)	0.0534 (7)	
H21A	0.2332	0.8571	0.3531	0.064*	
C22	−0.0769 (3)	0.7401 (2)	0.4407 (2)	0.0496 (6)	
F2	1.1717 (2)	0.0812 (2)	−0.19963 (18)	0.1277 (9)	
N4	0.93059 (18)	0.46094 (17)	0.16255 (15)	0.0425 (5)	
N5	1.12797 (19)	0.3232 (2)	0.24295 (18)	0.0627 (7)	
H5B	1.1201	0.3707	0.2799	0.075*	
H5C	1.1960	0.2556	0.2515	0.075*	
N6	1.2344 (2)	0.0654 (2)	0.1625 (2)	0.0689 (7)	
C23	0.6735 (2)	0.6982 (2)	−0.0081 (2)	0.0477 (6)	
H23A	0.7082	0.6797	−0.0683	0.057*	
C24	0.5719 (3)	0.8136 (2)	−0.0161 (2)	0.0598 (8)	
H24A	0.5402	0.8702	−0.0812	0.072*	
C25	0.5195 (3)	0.8432 (2)	0.0703 (2)	0.0582 (7)	
H25A	0.4539	0.9208	0.0639	0.070*	
C26	0.5635 (2)	0.7576 (2)	0.1697 (2)	0.0454 (6)	
C27	0.5086 (3)	0.7860 (3)	0.2608 (2)	0.0617 (8)	
H27A	0.4458	0.8646	0.2555	0.074*	
C28	0.5453 (3)	0.7015 (3)	0.3551 (2)	0.0676 (9)	
H28A	0.5096	0.7221	0.4142	0.081*	
C29	0.6378 (3)	0.5825 (3)	0.3629 (2)	0.0645 (8)	
H29A	0.6602	0.5231	0.4272	0.077*	
C30	0.6958 (2)	0.5522 (2)	0.27764 (19)	0.0483 (6)	
H30A	0.7575	0.4726	0.2850	0.058*	
C31	0.6637 (2)	0.6395 (2)	0.17865 (18)	0.0386 (6)	
C32	0.7222 (2)	0.6128 (2)	0.08634 (18)	0.0387 (6)	
C33	0.8349 (2)	0.4955 (2)	0.09160 (18)	0.0389 (6)	
C34	1.0336 (2)	0.3545 (2)	0.17145 (19)	0.0425 (6)	
C35	1.0429 (2)	0.2773 (2)	0.11042 (18)	0.0399 (6)	
C36	0.9455 (2)	0.3158 (2)	0.03418 (18)	0.0399 (6)	
C37	0.8406 (2)	0.4266 (2)	0.02531 (18)	0.0421 (6)	
H37A	0.7740	0.4553	−0.0246	0.050*	
C38	0.9551 (2)	0.2384 (2)	−0.03374 (18)	0.0453 (6)	
C39	1.0620 (3)	0.1951 (3)	−0.0867 (2)	0.0616 (8)	
H39A	1.1287	0.2146	−0.0818	0.074*	
C40	1.0671 (3)	0.1222 (3)	−0.1472 (2)	0.0746 (10)	
C41	0.9727 (4)	0.0891 (3)	−0.1559 (2)	0.0806 (11)	
H41A	0.9803	0.0381	−0.1961	0.097*	
C42	0.8678 (3)	0.1329 (3)	−0.1043 (2)	0.0752 (9)	
H42A	0.8017	0.1127	−0.1098	0.090*	
C43	0.8581 (3)	0.2075 (2)	−0.0435 (2)	0.0582 (7)	
H43A	0.7854	0.2371	−0.0087	0.070*	
C44	1.1486 (2)	0.1592 (2)	0.1355 (2)	0.0474 (6)	

### Atomic displacement parameters (Å^2^)

**Table:**

	*U*^11^	*U*^22^	*U*^33^	*U*^12^	*U*^13^	*U*^23^
F1	0.1427 (19)	0.1135 (16)	0.0613 (12)	−0.0636 (15)	0.0257 (12)	−0.0511 (11)
N1	0.0374 (11)	0.0473 (12)	0.0523 (13)	−0.0180 (10)	0.0051 (9)	−0.0224 (10)
N2	0.0424 (13)	0.0780 (17)	0.0986 (19)	−0.0288 (12)	0.0184 (13)	−0.0596 (15)
N3	0.0572 (16)	0.0741 (18)	0.098 (2)	−0.0262 (14)	0.0272 (15)	−0.0454 (16)
C1	0.0564 (16)	0.0697 (18)	0.0492 (16)	−0.0368 (15)	0.0082 (13)	−0.0258 (14)
C2	0.0676 (19)	0.103 (3)	0.0548 (18)	−0.056 (2)	0.0262 (15)	−0.0385 (18)
C3	0.0524 (17)	0.108 (3)	0.067 (2)	−0.0390 (19)	0.0276 (15)	−0.056 (2)
C4	0.0367 (14)	0.0717 (19)	0.0516 (16)	−0.0164 (14)	0.0064 (12)	−0.0337 (14)
C5	0.0493 (17)	0.080 (2)	0.076 (2)	−0.0056 (17)	0.0039 (16)	−0.0450 (19)
C6	0.067 (2)	0.0535 (18)	0.080 (2)	−0.0019 (16)	−0.0105 (18)	−0.0261 (17)
C7	0.0553 (17)	0.0534 (17)	0.0627 (18)	−0.0140 (14)	−0.0061 (14)	−0.0113 (14)
C8	0.0376 (13)	0.0531 (16)	0.0467 (15)	−0.0113 (12)	−0.0003 (11)	−0.0164 (13)
C9	0.0324 (12)	0.0549 (15)	0.0409 (14)	−0.0156 (12)	0.0005 (10)	−0.0225 (12)
C10	0.0365 (13)	0.0568 (16)	0.0388 (13)	−0.0213 (12)	0.0031 (11)	−0.0227 (12)
C11	0.0389 (13)	0.0420 (13)	0.0352 (13)	−0.0166 (11)	0.0027 (10)	−0.0117 (11)
C12	0.0379 (13)	0.0446 (14)	0.0537 (15)	−0.0176 (11)	0.0057 (11)	−0.0214 (12)
C13	0.0393 (13)	0.0401 (13)	0.0425 (14)	−0.0151 (11)	0.0051 (11)	−0.0168 (11)
C14	0.0462 (14)	0.0377 (13)	0.0347 (13)	−0.0181 (11)	0.0021 (11)	−0.0101 (10)
C15	0.0411 (14)	0.0488 (14)	0.0436 (14)	−0.0228 (12)	0.0038 (11)	−0.0171 (12)
C16	0.0420 (14)	0.0403 (13)	0.0418 (14)	−0.0145 (11)	−0.0016 (11)	−0.0147 (11)
C17	0.0574 (16)	0.0486 (15)	0.0450 (15)	−0.0231 (13)	0.0026 (12)	−0.0172 (12)
C18	0.0671 (19)	0.0623 (18)	0.0479 (16)	−0.0238 (16)	0.0033 (14)	−0.0285 (14)
C19	0.071 (2)	0.0639 (19)	0.073 (2)	−0.0287 (17)	−0.0039 (16)	−0.0372 (16)
C20	0.073 (2)	0.0658 (19)	0.082 (2)	−0.0422 (17)	0.0104 (17)	−0.0306 (17)
C21	0.0554 (16)	0.0591 (17)	0.0560 (17)	−0.0307 (14)	0.0082 (13)	−0.0233 (14)
C22	0.0518 (16)	0.0493 (15)	0.0574 (16)	−0.0255 (14)	0.0103 (13)	−0.0257 (13)
F2	0.1271 (19)	0.138 (2)	0.1066 (17)	−0.0206 (16)	0.0424 (15)	−0.0871 (16)
N4	0.0350 (11)	0.0414 (11)	0.0517 (12)	−0.0109 (9)	0.0019 (9)	−0.0240 (10)
N5	0.0414 (12)	0.0647 (15)	0.0767 (16)	−0.0025 (11)	−0.0149 (11)	−0.0436 (13)
N6	0.0590 (15)	0.0497 (15)	0.0838 (18)	−0.0059 (13)	−0.0038 (13)	−0.0270 (13)
C23	0.0495 (15)	0.0469 (15)	0.0446 (15)	−0.0185 (13)	0.0034 (12)	−0.0153 (12)
C24	0.0603 (18)	0.0463 (16)	0.0525 (17)	−0.0116 (14)	−0.0051 (14)	−0.0031 (13)
C25	0.0503 (16)	0.0379 (14)	0.0666 (19)	−0.0030 (12)	0.0010 (14)	−0.0133 (14)
C26	0.0385 (13)	0.0420 (14)	0.0544 (16)	−0.0126 (11)	0.0036 (12)	−0.0207 (12)
C27	0.0443 (16)	0.0639 (18)	0.073 (2)	−0.0101 (14)	0.0071 (14)	−0.0374 (17)
C28	0.0502 (17)	0.093 (2)	0.0523 (18)	−0.0161 (17)	0.0083 (14)	−0.0379 (18)
C29	0.0481 (16)	0.083 (2)	0.0451 (16)	−0.0177 (16)	−0.0003 (13)	−0.0117 (15)
C30	0.0336 (13)	0.0509 (15)	0.0489 (15)	−0.0092 (12)	−0.0015 (11)	−0.0129 (13)
C31	0.0304 (12)	0.0410 (13)	0.0454 (14)	−0.0134 (11)	0.0003 (10)	−0.0177 (11)
C32	0.0357 (13)	0.0368 (13)	0.0458 (14)	−0.0142 (11)	0.0011 (11)	−0.0176 (11)
C33	0.0342 (12)	0.0389 (13)	0.0426 (14)	−0.0132 (11)	0.0026 (11)	−0.0154 (11)
C34	0.0375 (13)	0.0471 (14)	0.0455 (14)	−0.0148 (12)	0.0023 (11)	−0.0236 (12)
C35	0.0377 (13)	0.0379 (13)	0.0431 (14)	−0.0122 (11)	0.0071 (11)	−0.0186 (11)
C36	0.0425 (14)	0.0399 (13)	0.0371 (13)	−0.0150 (11)	0.0057 (11)	−0.0167 (11)
C37	0.0414 (13)	0.0442 (14)	0.0425 (14)	−0.0158 (11)	−0.0006 (11)	−0.0197 (11)
C38	0.0531 (15)	0.0389 (13)	0.0366 (13)	−0.0103 (12)	−0.0023 (11)	−0.0160 (11)
C39	0.0697 (19)	0.0598 (17)	0.0519 (16)	−0.0189 (15)	0.0097 (14)	−0.0280 (14)
C40	0.087 (2)	0.0625 (19)	0.0509 (18)	−0.0042 (18)	0.0111 (17)	−0.0318 (16)
C41	0.115 (3)	0.0545 (19)	0.059 (2)	−0.013 (2)	−0.019 (2)	−0.0321 (16)
C42	0.092 (2)	0.0621 (19)	0.074 (2)	−0.0260 (18)	−0.0182 (19)	−0.0328 (18)
C43	0.0658 (18)	0.0546 (17)	0.0582 (17)	−0.0222 (15)	−0.0026 (14)	−0.0279 (14)
C44	0.0464 (15)	0.0452 (15)	0.0530 (16)	−0.0164 (13)	0.0060 (12)	−0.0243 (13)

### Geometric parameters (Å, °)

**Table:**

F1—C18	1.363 (3)	F2—C40	1.354 (3)
N1—C12	1.336 (3)	N4—C34	1.339 (3)
N1—C11	1.341 (3)	N4—C33	1.347 (3)
N2—C12	1.352 (3)	N5—C34	1.351 (3)
N2—H2A	0.8600	N5—H5B	0.8600
N2—H2B	0.8600	N5—H5C	0.8600
N3—C22	1.144 (3)	N6—C44	1.143 (3)
C1—C10	1.364 (3)	C23—C32	1.366 (3)
C1—C2	1.403 (4)	C23—C24	1.405 (4)
C1—H1B	0.9300	C23—H23A	0.9300
C2—C3	1.352 (4)	C24—C25	1.357 (4)
C2—H2C	0.9300	C24—H24A	0.9300
C3—C4	1.409 (4)	C25—C26	1.410 (4)
C3—H3A	0.9300	C25—H25A	0.9300
C4—C5	1.411 (4)	C26—C27	1.418 (3)
C4—C9	1.420 (3)	C26—C31	1.419 (3)
C5—C6	1.351 (4)	C27—C28	1.349 (4)
C5—H5A	0.9300	C27—H27A	0.9300
C6—C7	1.394 (4)	C28—C29	1.398 (4)
C6—H6A	0.9300	C28—H28A	0.9300
C7—C8	1.362 (4)	C29—C30	1.365 (3)
C7—H7A	0.9300	C29—H29A	0.9300
C8—C9	1.407 (3)	C30—C31	1.410 (3)
C8—H8A	0.9300	C30—H30A	0.9300
C9—C10	1.428 (3)	C31—C32	1.440 (3)
C10—C11	1.486 (3)	C32—C33	1.483 (3)
C11—C15	1.394 (3)	C33—C37	1.398 (3)
C12—C13	1.423 (3)	C34—C35	1.416 (3)
C13—C14	1.392 (3)	C35—C36	1.397 (3)
C13—C22	1.430 (3)	C35—C44	1.425 (3)
C14—C15	1.388 (3)	C36—C37	1.383 (3)
C14—C16	1.488 (3)	C36—C38	1.491 (3)
C15—H15A	0.9300	C37—H37A	0.9300
C16—C21	1.380 (3)	C38—C43	1.381 (4)
C16—C17	1.399 (3)	C38—C39	1.381 (4)
C17—C18	1.367 (4)	C39—C40	1.380 (4)
C17—H17A	0.9300	C39—H39A	0.9300
C18—C19	1.350 (4)	C40—C41	1.369 (5)
C19—C20	1.387 (4)	C41—C42	1.355 (4)
C19—H19A	0.9300	C41—H41A	0.9300
C20—C21	1.383 (4)	C42—C43	1.386 (4)
C20—H20A	0.9300	C42—H42A	0.9300
C21—H21A	0.9300	C43—H43A	0.9300
			
C12—N1—C11	118.2 (2)	C34—N4—C33	118.28 (19)
C12—N2—H2A	120.0	C34—N5—H5B	120.0
C12—N2—H2B	120.0	C34—N5—H5C	120.0
H2A—N2—H2B	120.0	H5B—N5—H5C	120.0
C10—C1—C2	121.5 (3)	C32—C23—C24	121.1 (2)
C10—C1—H1B	119.2	C32—C23—H23A	119.4
C2—C1—H1B	119.2	C24—C23—H23A	119.4
C3—C2—C1	119.8 (3)	C25—C24—C23	120.7 (2)
C3—C2—H2C	120.1	C25—C24—H24A	119.6
C1—C2—H2C	120.1	C23—C24—H24A	119.6
C2—C3—C4	121.5 (3)	C24—C25—C26	120.7 (2)
C2—C3—H3A	119.3	C24—C25—H25A	119.7
C4—C3—H3A	119.3	C26—C25—H25A	119.7
C3—C4—C5	121.8 (3)	C25—C26—C27	121.6 (2)
C3—C4—C9	118.8 (3)	C25—C26—C31	119.2 (2)
C5—C4—C9	119.3 (3)	C27—C26—C31	119.2 (2)
C6—C5—C4	120.8 (3)	C28—C27—C26	121.4 (3)
C6—C5—H5A	119.6	C28—C27—H27A	119.3
C4—C5—H5A	119.6	C26—C27—H27A	119.3
C5—C6—C7	120.2 (3)	C27—C28—C29	119.3 (3)
C5—C6—H6A	119.9	C27—C28—H28A	120.3
C7—C6—H6A	119.9	C29—C28—H28A	120.3
C8—C7—C6	120.6 (3)	C30—C29—C28	121.1 (3)
C8—C7—H7A	119.7	C30—C29—H29A	119.4
C6—C7—H7A	119.7	C28—C29—H29A	119.4
C7—C8—C9	121.1 (2)	C29—C30—C31	121.1 (2)
C7—C8—H8A	119.4	C29—C30—H30A	119.4
C9—C8—H8A	119.4	C31—C30—H30A	119.4
C8—C9—C4	117.8 (2)	C30—C31—C26	117.5 (2)
C8—C9—C10	123.4 (2)	C30—C31—C32	123.5 (2)
C4—C9—C10	118.8 (2)	C26—C31—C32	118.9 (2)
C1—C10—C9	119.5 (2)	C23—C32—C31	119.2 (2)
C1—C10—C11	118.4 (2)	C23—C32—C33	119.3 (2)
C9—C10—C11	122.1 (2)	C31—C32—C33	121.5 (2)
N1—C11—C15	122.5 (2)	N4—C33—C37	122.6 (2)
N1—C11—C10	116.8 (2)	N4—C33—C32	116.09 (19)
C15—C11—C10	120.6 (2)	C37—C33—C32	121.3 (2)
N1—C12—N2	116.7 (2)	N4—C34—N5	116.6 (2)
N1—C12—C13	122.2 (2)	N4—C34—C35	122.0 (2)
N2—C12—C13	121.1 (2)	N5—C34—C35	121.4 (2)
C14—C13—C12	119.4 (2)	C36—C35—C34	119.4 (2)
C14—C13—C22	123.2 (2)	C36—C35—C44	123.1 (2)
C12—C13—C22	117.2 (2)	C34—C35—C44	117.4 (2)
C15—C14—C13	117.1 (2)	C37—C36—C35	117.7 (2)
C15—C14—C16	120.7 (2)	C37—C36—C38	121.8 (2)
C13—C14—C16	122.2 (2)	C35—C36—C38	120.5 (2)
C14—C15—C11	120.5 (2)	C36—C37—C33	119.9 (2)
C14—C15—H15A	119.8	C36—C37—H37A	120.1
C11—C15—H15A	119.8	C33—C37—H37A	120.1
C21—C16—C17	118.9 (2)	C43—C38—C39	119.1 (2)
C21—C16—C14	120.2 (2)	C43—C38—C36	120.5 (2)
C17—C16—C14	120.9 (2)	C39—C38—C36	120.4 (2)
C18—C17—C16	118.8 (3)	C40—C39—C38	118.1 (3)
C18—C17—H17A	120.6	C40—C39—H39A	120.9
C16—C17—H17A	120.6	C38—C39—H39A	120.9
C19—C18—F1	118.0 (2)	F2—C40—C41	119.0 (3)
C19—C18—C17	123.5 (3)	F2—C40—C39	117.7 (4)
F1—C18—C17	118.4 (3)	C41—C40—C39	123.3 (3)
C18—C19—C20	117.7 (3)	C42—C41—C40	118.1 (3)
C18—C19—H19A	121.1	C42—C41—H41A	121.0
C20—C19—H19A	121.1	C40—C41—H41A	121.0
C21—C20—C19	120.8 (3)	C41—C42—C43	120.5 (3)
C21—C20—H20A	119.6	C41—C42—H42A	119.7
C19—C20—H20A	119.6	C43—C42—H42A	119.7
C16—C21—C20	120.2 (3)	C38—C43—C42	120.9 (3)
C16—C21—H21A	119.9	C38—C43—H43A	119.6
C20—C21—H21A	119.9	C42—C43—H43A	119.6
N3—C22—C13	175.7 (3)	N6—C44—C35	175.1 (3)
			
C10—C1—C2—C3	−0.9 (4)	C32—C23—C24—C25	0.1 (4)
C1—C2—C3—C4	−0.8 (4)	C23—C24—C25—C26	−1.8 (4)
C2—C3—C4—C5	−178.9 (3)	C24—C25—C26—C27	−178.9 (3)
C2—C3—C4—C9	0.7 (4)	C24—C25—C26—C31	0.0 (4)
C3—C4—C5—C6	179.5 (3)	C25—C26—C27—C28	175.7 (3)
C9—C4—C5—C6	−0.1 (4)	C31—C26—C27—C28	−3.2 (4)
C4—C5—C6—C7	−1.6 (5)	C26—C27—C28—C29	−1.3 (5)
C5—C6—C7—C8	1.5 (5)	C27—C28—C29—C30	3.2 (5)
C6—C7—C8—C9	0.4 (4)	C28—C29—C30—C31	−0.5 (4)
C7—C8—C9—C4	−2.1 (4)	C29—C30—C31—C26	−3.9 (4)
C7—C8—C9—C10	179.3 (2)	C29—C30—C31—C32	179.6 (2)
C3—C4—C9—C8	−177.6 (2)	C25—C26—C31—C30	−173.3 (2)
C5—C4—C9—C8	2.0 (4)	C27—C26—C31—C30	5.6 (3)
C3—C4—C9—C10	1.0 (3)	C25—C26—C31—C32	3.4 (3)
C5—C4—C9—C10	−179.4 (2)	C27—C26—C31—C32	−177.7 (2)
C2—C1—C10—C9	2.6 (4)	C24—C23—C32—C31	3.4 (4)
C2—C1—C10—C11	−175.8 (2)	C24—C23—C32—C33	−176.0 (2)
C8—C9—C10—C1	175.9 (2)	C30—C31—C32—C23	171.4 (2)
C4—C9—C10—C1	−2.6 (3)	C26—C31—C32—C23	−5.1 (3)
C8—C9—C10—C11	−5.8 (4)	C30—C31—C32—C33	−9.3 (3)
C4—C9—C10—C11	175.7 (2)	C26—C31—C32—C33	174.3 (2)
C12—N1—C11—C15	1.4 (4)	C34—N4—C33—C37	−1.9 (3)
C12—N1—C11—C10	−176.9 (2)	C34—N4—C33—C32	178.9 (2)
C1—C10—C11—N1	123.8 (2)	C23—C32—C33—N4	131.0 (2)
C9—C10—C11—N1	−54.6 (3)	C31—C32—C33—N4	−48.3 (3)
C1—C10—C11—C15	−54.6 (3)	C23—C32—C33—C37	−48.2 (3)
C9—C10—C11—C15	127.1 (3)	C31—C32—C33—C37	132.4 (2)
C11—N1—C12—N2	176.6 (2)	C33—N4—C34—N5	179.6 (2)
C11—N1—C12—C13	−4.0 (4)	C33—N4—C34—C35	−1.4 (4)
N1—C12—C13—C14	3.4 (4)	N4—C34—C35—C36	3.9 (4)
N2—C12—C13—C14	−177.2 (2)	N5—C34—C35—C36	−177.1 (2)
N1—C12—C13—C22	179.5 (2)	N4—C34—C35—C44	−171.9 (2)
N2—C12—C13—C22	−1.1 (4)	N5—C34—C35—C44	7.1 (4)
C12—C13—C14—C15	−0.1 (3)	C34—C35—C36—C37	−3.0 (3)
C22—C13—C14—C15	−175.9 (2)	C44—C35—C36—C37	172.5 (2)
C12—C13—C14—C16	179.2 (2)	C34—C35—C36—C38	177.8 (2)
C22—C13—C14—C16	3.3 (4)	C44—C35—C36—C38	−6.7 (4)
C13—C14—C15—C11	−2.4 (3)	C35—C36—C37—C33	−0.1 (3)
C16—C14—C15—C11	178.4 (2)	C38—C36—C37—C33	179.1 (2)
N1—C11—C15—C14	1.8 (4)	N4—C33—C37—C36	2.7 (4)
C10—C11—C15—C14	−179.9 (2)	C32—C33—C37—C36	−178.2 (2)
C15—C14—C16—C21	46.0 (3)	C37—C36—C38—C43	−51.8 (3)
C13—C14—C16—C21	−133.2 (3)	C35—C36—C38—C43	127.4 (3)
C15—C14—C16—C17	−132.4 (3)	C37—C36—C38—C39	129.1 (3)
C13—C14—C16—C17	48.4 (3)	C35—C36—C38—C39	−51.7 (3)
C21—C16—C17—C18	−0.9 (4)	C43—C38—C39—C40	−0.2 (4)
C14—C16—C17—C18	177.5 (2)	C36—C38—C39—C40	179.0 (3)
C16—C17—C18—C19	1.5 (4)	C38—C39—C40—F2	179.4 (3)
C16—C17—C18—F1	−178.9 (2)	C38—C39—C40—C41	−1.1 (5)
F1—C18—C19—C20	179.7 (3)	F2—C40—C41—C42	−178.8 (3)
C17—C18—C19—C20	−0.7 (5)	C39—C40—C41—C42	1.6 (5)
C18—C19—C20—C21	−0.7 (5)	C40—C41—C42—C43	−0.9 (5)
C17—C16—C21—C20	−0.5 (4)	C39—C38—C43—C42	0.8 (4)
C14—C16—C21—C20	−178.8 (3)	C36—C38—C43—C42	−178.3 (3)
C19—C20—C21—C16	1.3 (4)	C41—C42—C43—C38	−0.3 (5)
C14—C13—C22—N3	161 (4)	C36—C35—C44—N6	−156 (3)
C12—C13—C22—N3	−15 (4)	C34—C35—C44—N6	19 (3)

### Hydrogen-bond geometry (Å, °)

**Table:**

*D*—H···*A*	*D*—H	H···*A*	*D*···*A*	*D*—H···*A*
N2—H2A···N4^i^	0.86	2.30	3.108 (3)	157
N5—H5B···N1^ii^	0.86	2.25	3.031 (3)	152
C8—H8A···N1	0.93	2.59	3.094 (3)	114
C30—H30A···N4	0.93	2.53	3.017 (3)	113

Symmetry codes: (i) *x*−1, *y*, *z*; (ii) *x*+1, *y*, *z*.
